# Synergistic Interaction of Methanol Extract from* Canarium odontophyllum* Miq. Leaf in Combination with Oxacillin against Methicillin-Resistant* Staphylococcus aureus* (MRSA) ATCC 33591

**DOI:** 10.1155/2016/5249534

**Published:** 2016-02-23

**Authors:** Dayang Fredalina Basri, Vimashiinee Sandra

**Affiliations:** School of Diagnostic & Applied Health Sciences, Faculty of Health Sciences, Universiti Kebangsaan Malaysia, Jalan Raja Muda Abdul Aziz, 50300 Kuala Lumpur, Malaysia

## Abstract

*Canarium odontophyllum* (CO) Miq. has been considered as one of the most sought-after plant species in Sarawak, Malaysia, due to its nutritional and pharmacological benefits. This study aimed to evaluate the pharmacodynamic interaction of crude methanol and acetone extracts from CO leaves in combination with oxacillin, vancomycin, and linezolid, respectively, against MRSA ATCC 33591 as preliminary study has reported its potential antistaphylococcal activity. The broth microdilution assay revealed that both methanol and acetone extracts were bactericidal with Minimum Inhibitory Concentration (MIC) of 312.5 *μ*g/mL and 156.25 *μ*g/mL and Minimum Bactericidal Concentration (MBC) of 625 *μ*g/mL and 312.5 *μ*g/mL, respectively. Fractional Inhibitory Concentration (FIC) indices were obtained via the chequerboard dilution assay where methanol extract-oxacillin, acetone extract-oxacillin, methanol extract-linezolid, and acetone extract-linezolid combinations exhibited synergism (FIC index ≤ 0.5). The synergistic action of the methanol extract-oxacillin combination was verified by time-kill analysis where bactericidal effect was observed at concentration of 1/8 × MIC of both compounds at 9.6 h compared to oxacillin alone. As such, these findings postulated that both extracts exert their anti-MRSA mechanism of action similar to that of vancomycin and provide evidence that the leaves of* C. odontophyllum* have the potential to be developed into antistaphylococcal agents.

## 1. Introduction

MRSA has been a major cause of community, endemic, and epidemic nosocomial infections [1]. MRSA infections cause a range of illnesses, from skin and wound infection to pneumonia and blood stream infections that can cause sepsis and death. The pathogen poses a huge threat as it has started to show resistance towards the last line of antibiotic treatment for Gram-positive bacteria [2–5] with vancomycin-resistant* S. aureus* [6] and linezolid-resistant* S. aureus* (LRSA) strains being reported worldwide [7]. Natural products, especially plants, have always been a great source of biological compounds for medicinal purposes [8]. Plants and their secondary metabolites offer a diverse reservoir of biologically active components as potentially therapeutic agents, including antimicrobials [9]. These compounds are naturally present to protect plants from microorganisms, insects, and herbivores as well as giving plants their odour and pigments. Studies have revealed that many medicinal plant species from around the world can provide alternatives for a wide range of bacterial infections [10].* Canarium odontophyllum* Miq., locally known as “dabai,” is a very popular fruit in Sarawak, largely consumed by the locals during its season. It is native to the tropical rainforest of Borneo and is reported as one of the underutilised fruits of Sarawak. The CO fruit is rich in unsaturated fatty acids and contains sixteen types of phenolic compounds as well as exhibiting great potential as antioxidant [11–13]. Preliminary screening [14, 15] of methanol and acetone extracts of CO leaves showed antimicrobial activity towards* Staphylococcus aureus* and MRSA.

Natural products may be in itself toxic to the microorganism or be a resistance-modifying agent in the microorganism such as clavulinic acid, a beta-lactamase inhibitor in Augmentin. Experiments studying the combinatory effects of drug and natural compound may lead to discovery of a novel therapeutic agent and hamper the development of microbial resistance towards antibiotic. Interaction between existing drug and natural product may exert different mechanisms of action against the same strain of microorganism. Synergistic effect of phytocompounds with antibiotics consequently results in lesser dose of antibiotic required for treatment [16, 17]. Synergism is defined as the type of interaction where combination of two different compounds produces greater activity than either compound alone. Indifference means that the combination produced no additional effect, either positive or negative, and is no different than either compound alone. Additive interaction is seen as a slight improvement in activity as if the concentration of either compound has been increased. Antagonism, on the other hand, indicates that the combination results in worsening effects compared to either compound alone [18–20].

*S. aureus* especially MRSA is relatively ubiquitous and is generally resistant to many chemotherapeutic agents including vancomycin, hence the cornerstone of treatment against MRSA infection [7]. Therefore, sourcing alternative antimicrobial agents is crucial and, currently, is a global challenge. Natural products and their derivatives have continued to be the most significant source of new lead into the development of new pharmaceutical agent. The present study, therefore, aimed to evaluate the anti-MRSA activity of methanol and acetone extracts from CO leaves in combination with three selected antimicrobial agents, namely, oxacillin, vancomycin, and linezolid. The antimicrobial susceptibility of MRSA towards CO leaves extract was analyzed via the broth microdilution method.

## 2. Materials and Methods

### 2.1. Chemicals and Reagents

Absolute methanol and absolute acetone, respectively, were used to prepare the methanol and acetone extracts of CO leaves. Dimethyl sulfoxide (DMSO) was used as diluting solvent for linezolid. Triphenyl tetrazolium chloride (TTC) was used as an indicator of metabolically active cells in the Minimum Inhibitory Concentration (MIC) and fractional inhibitory concentration (FIC) tests. Crystallized methanol and acetone extracts of CO leaves were tested in this study. Antimicrobial agents, oxacillin, vancomycin, and linezolid, used in this study were obtained from Sigma Aldrich (USA).

### 2.2. Growth, Suspension, and Storage Media

The bacterial strain was grown and maintained on nutrient agar slants. Nutrient agar plates were used to culture the bacteria prior to suspension preparation. Mueller-Hinton broth (MHB) was used to prepare the bacterial suspension and Mueller-Hinton agar (MHA) was used to culture bacteria from the broth microdilution plate for determination of minimal bactericidal concentration (MBC).

### 2.3. Plant Material

The leaf of CO was obtained from Sarawak, Malaysia. All plant parts were identified and authenticated by Mr. Sani Miran and deposited in the Herbarium of the Universiti Kebangsaan Malaysia (UKM), Bangi, Selangor, Malaysia, with a voucher specimen number of UKMB 40052. The whole leaf was used for preparation of the extracts.

### 2.4. Preparation of Extracts and Antimicrobial Agents

The stock solutions of the test material were prepared by dissolving the methanol and acetone extracts of CO leaves in absolute methanol and absolute acetone, respectively, to a final concentration of 100 mg/mL and stored at 4°C for subsequent use. The stock solutions of oxacillin and vancomycin were prepared by dissolving in sterile distilled water while linezolid was dissolved in 10% DMSO to a final concentration of 100 mg/mL. The working solutions of the tested extracts and the antimicrobial agents were prepared by calculating the twofold dilution factor of the stock solution and sterilised by filtration through millipore membrane filter of 0.45 *μ*m pore size.

### 2.5. Preparation of Bacterial Inoculum

Isolated single colony from the bacterial culture was selected, inoculated into MHB, and incubated at 37°C. The inoculum size for the MIC, MBC, and FIC tests was standardized to 10^6^ CFU/mL by adjusting the optical density of the bacterial suspension to a turbidity corresponding to spectrophotometric absorbance of 0.08 to 0.13 at 620 nm. This reading is comparable to that of 0.5 McFarland standard which is equivalent to a bacterial count of approximately 10^8^ CFU/mL followed by 1 : 100 dilution to produce a bacterial concentration of 10^6^ CFU/mL [21].

### 2.6. Determination of MIC and MBC

Both CO extracts and the three antibiotics were tested to determine their MIC values via the broth microdilution method based on Clinical Laboratory Standards Institute guidelines [21] as adapted by Basri and Khairon [22]. The MIC assays were carried out in 96-well microtitre plates in triplicate at a twofold serial dilution of the tested compounds from 9.77 *μ*g/mL to 5,000 *μ*g/mL for the extracts and from 0.19 *μ*g/mL to 2,000 *μ*g/mL for antibiotics. Then bacterial suspension was added such that the bacterial concentration in each well is 5 × 10^5^ CFU/mL. Negative control comprised MHB and extracts or antibiotics while the positive control was MHB and bacterial suspension only. The plates were then incubated at 37°C for 18 to 24 h. After incubation, the plates were visually examined for bacterial growth. From each well that showed no visible growth, samples were subcultured on sterile Mueller-Hinton agar plates to determine the MBC value. The plates were then incubated at 37°C for 24 h. The well containing the lowest concentration of the compound with no visible bacterial growth was taken as the MIC value. This is further validated by addition of triphenyl tetrazolium chloride (TTC) to the wells. TTC with a concentration of 2 mg/mL and a volume of 20 *μ*L was added to each well and incubated for 20 min. Wells that appear pink comparable to that of the positive control were interpreted as positive for bacterial growth while wells with colourless solution were interpreted as negative for bacterial growth. The well containing the lowest concentration of the colourless solution was interpreted as the MIC. The MBC was interpreted as lowest concentration showing no visible growth on agar subculture.

### 2.7. Determination of FIC Index

The combined effect of methanol extract and acetone extract with the three selected antibiotics was evaluated by chequerboard dilution method [23] from which the fractional inhibitory concentration (FIC) index, the predictor of the type of interaction between compounds, was obtained [24]. The extracts were individually tested in combination with each of the three antibiotics separately on 96-well microtitre plates in triplicate. The concentrations tested for every combination of extract-antibiotic were their respective MICs, followed by 1/2, 1/4, 1/8, and 1/16 times of their respective MICs. The extract-antibiotic combination was added in 1 : 1 ratio in concentrations accordingly to wells that have already been filled with MHB. The bacterial suspension was added to the wells such that the bacterial concentration is 5 × 10^5^ CFU/mL in each well. Negative controls were MHB and the extract-antibiotic combination while positive controls were MHB and bacterial suspension. The plates were then incubated at 37°C for 24 h. Lastly, 20 *μ*L of TTC (2 mg/mL) was added to each well and the plates were incubated again for 20 min. Wells containing the solution which turned pink comparable to that of the positive control were interpreted as positive for bacterial growth. The wells containing solutions that remained colourless were interpreted as negative for bacterial growth. The assay was run in six replicates where an agreement between five or more replicates was required for estimation of FIC [25]. The FIC indices for the combination with negative results were calculated using the following formula [26]:

(1) $$\begin{array}{c} \frac{A}{{MIC}_{A}}+\frac{B}{{MIC}_{B}}={FIC}_{A}+{FIC}_{B}=\text{FIC Index}, \end{array}$$

where A is the MIC of compound A in combination, B is the MIC of compound B in combination, MIC_A_ is the MIC of compound A alone, and MIC_B_ is the MIC of compound B alone.

FIC index of 0.5 or less has traditionally been defined as synergism. FIC index between 0.5 and 2.0 is defined as additive and between 2.0 and 4.0, as indifference. FIC index more than 4.0 is defined as antagonism [27].

### 2.8. Time-Kill Analysis

The extract-antibiotic combination which has shown synergism with the lowest FIC index was further analyzed using the time-kill assay. Samples containing extract and antibiotic in concentrations identical to that of the synergistic combination were prepared. Bacterial suspension of concentration 10^8^ CFU/mL was added to the tubes such that the final bacterial concentration is 5 × 10^7^ CFU/mL. The tubes were then incubated at 37°C. At zero hour, 10 *μ*L of the sample was drawn from one of the tubes and diluted following a tenfold serial dilution in normal saline (0.9% NaCl). Next, 10 *μ*L from each of the dilution was cultured on Mueller-Hinton agar (MHA) plates and incubated at 37°C for 24 h. After incubation, colony count of the bacterial culture was performed. The number of colonies between 30 and 300 was taken into account for calculation of bacterial concentration at that hour. The entire process was performed at 4, 8, 12, and 24 h. A time-mortality curve was constructed with time (hour) along the *x*-axis and log_10_⁡ of bacterial concentration (CFU/mL) along the *y*-axis [28]. The results are expressed as the mean ± standard error of the mean (S.E.M) in the chart. The extract and antibiotic alone at their MIC concentrations were negative controls while growth control with MHB and bacteria only was the positive control.

The interaction was interpreted as synergistic with a decrease of ≥2 log⁡ CFU/mL after 24 h in bacterial concentration compared to the most active single agent. Additive or indifference is described as a <2 log⁡ CFU/mL change in bacterial concentration after 24 h for the combination compared to the single, most active compound. Antagonism is defined as a ≥2 log⁡ CFU/mL increase in bacterial concentration after 24 h by the combination in comparison to the most active single compound. The compound would be considered bactericidal if it produced a 3log⁡ CFU/mL reduction in colony count during incubation period which denotes >99.9% killing [29].

## 3. Results

The antibacterial activity of methanol and acetone extracts of CO leaves against MRSA was evaluated using the broth microdilution method. Concentrations tested were between 5000 *μ*g/mL and 9.77 *μ*g/mL.  Table 1 shows that the MIC value of methanol extract was 312.5 *μ*g/mL, which was twice the MIC of acetone extract at 156.25 *μ*g/mL. The MBC of methanol extract is 625 *μ*g/mL, which is twice the value of its MBC. The MIC of acetone extract was 156.25 *μ*g/mL being equivalent to its MIC.

The FIC indices for each combination were calculated based on the MIC values shown in Tables 1 and 2. Four out of six extract-antimicrobial agent combinations exhibited synergism while the other two combinations showed additivity (Table 2). The methanol extract-oxacillin combination demonstrated the lowest FIC index, 0.25, while the acetone extract-oxacillin combination gave an FIC index of 0.375. Both combinations showed a reduction of oxacillin concentration by eightfold. The methanol extract-linezolid combination and acetone extract-linezolid combination showed borderline synergism with FIC index of 0.5 with a reduction of linezolid concentration by fourfold. However, the methanol extract-vancomycin and acetone extract-vancomycin combinations showed additivity with FIC index of 1.0625 for both.

**Figure 1:**
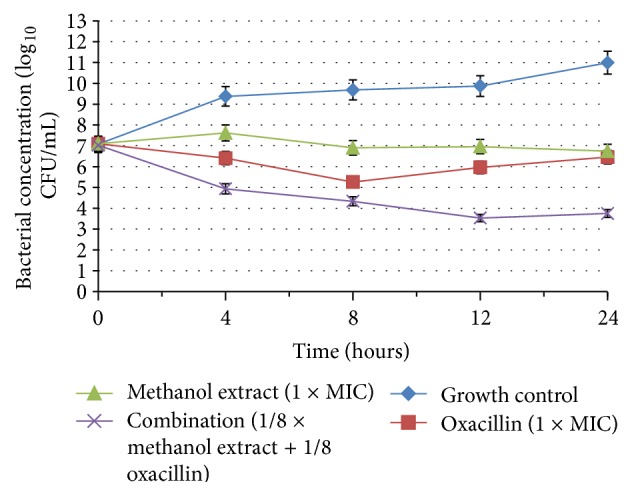
Chart shows the time-kill curve for the growth control, oxacillin, methanol extract, and methanol extract-oxacillin combination.

The time-kill assay of the methanol extract-oxacillin combination validated the findings of checkerboard microdilution test which produced an FIC index of 0.25 for the combination. The growth control was MHB and bacteria only. The methanol extract and oxacillin alone at their MIC were tested as negative control and for comparison with the combination. The time-kill curve is shown in Figure 1.

The synergistic effect of the methanol extract-oxacillin combination is apparent from the chart. In addition, a marked reduction of >2 log_10_⁡ CFU/mL was observed in growth of MRSA at 4 h for the combination compared with the extract or antibiotic alone. Moreover, the combination appeared to be bactericidal with a reduction of 3 log_10_⁡ CFU/mL at 9.6 h interval, which was neither seen with the extract or oxacillin. However, the curve showed a slight increase in bacterial concentration from 12 h to 24 h.

The methanol extract alone at its MIC showed a slight increase in bacterial concentration in the first four hours followed by a slow decrease till 24 h. Oxacillin, on the other hand, showed a decrease in bacterial concentration in first 8 h and subsequently increased until 24 h. Both compounds did not display bactericidal effect.

## 4. Discussions

The broth microdilution method is not only effective for quantification of bioactivity [30] but at the same time is able to directly measure the MIC [2]. Furthermore, the broth microdilution method has increased sensitivity for low quantity of extract, provides qualitative analysis of bacteriostatic and bacteriocidal effect, can be used to test on various microorganisms, and presents reproducible result [30].

Antimicrobials with MBC value being equal to or less than one dilution above their MIC is defined as bactericidal [31, 32]. The methanol and acetone extracts of* C. odontophyllum* leaves were both bactericidal, which is consistent with the findings reported in a previous study [15]. The standard antibiotics tested showed MIC consistent with the susceptibility range for each antimicrobial agent [33–36].

A phytochemical screening of CO leaf extracts revealed the phytoconstituents in aqueous, methanol, and acetone extracts. The components in methanol and acetone were identical as they consist of phenolic compound, flavonoid, terpenoid, and saponin. This raises the possibility that saponin is the key player in antibacterial activity in methanol and acetone extracts of CO leaves. Saponin extracts have been reported to exert antibacterial activity towards* S. aureus*,* E. coli*, and* P. aeruginosa* with* S. aureus* being the most susceptible bacteria [37, 38].

Four out of six extract-antimicrobial agent combinations displayed synergistic interaction and out of these four the methanol extract-oxacillin combination showed an eightfold reduction of oxacillin concentration. This combination demonstrated bactericidal activity with the rate-killing time at 9.6 h indicating that the lesser concentration of the compound produces an enhanced effect in combination which was greater than either compound alone.

Interaction of natural products with modern drugs is based on the same pharmacodynamic principles as drug-drug interaction [39]. When antimicrobial compounds are combined in laboratory conditions, they are found to interact in a few ways, namely, indifference, additive, synergism, or antagonism.

Synergism between plant extracts and standard antibiotics has been previously reported. Lemon grass oil, yacon leaf extracts, and tea extracts have all been reported to exert mutual antagonism in combination with beta-lactam antibiotics [40–42]. Plant secondary metabolites exist as complexes in the crude extracts and provide a wide range of chemical functional groups which bind to active sites on the target pathogens. Such association determines their antimicrobial activity and their efficacy is based on the combined action of a mixture of constituents [43, 44].

An interesting point to note is that the extracts produced synergistic effects with one of the cell wall inhibitors (oxacillin) and a protein synthesis inhibitor (linezolid). Despite that fact that both oxacillin and vancomycin are cell wall inhibitors, the extracts show synergism with oxacillin but additivity with vancomycin. Synergistic interactions occur when two compounds act against the target in different mechanism [45, 46], while additivity occurs when different compounds exert the same mechanism of action against the target. Thus, the notion that the extracts of CO leaves demonstrate antibacterial activity by inhibiting cell wall synthesis similar to the action of vancomycin can be put forward. A study has proven that cell wall inhibitors, beta lactams, and glycopeptides exert mutual antagonism against MRSA [47].

Healthcare professionals have used combination therapies to overcome antibiotic resistance in the clinical setting for the benefit of patient with multidrug-resistant infection. The same strategy could be employed with a slight change where plant secondary metabolites are used in combination with existing antimicrobial agents to produce a new antimicrobial agent. Combination of antibacterial agents that demonstrate synergism, partial synergism, and additivity could potentially improve clinical outcome of patient with infections that are difficult to treat [48].

## 5. Conclusion

These findings postulated that both methanol and acetone extracts from the leaves of* Canarium odontophyllum* exert synergistic interaction with oxacillin and linezolid with eightfold reduction in the MIC of oxacillin by methanol extract. The methanol extract in combination with oxacillin exhibited bactericidal action which was not observed individually. In addition, it can be postulated from this finding that the mechanism of action of CO leaves extracts was similar to that of vancomycin and provides evidence that the extracts have the potential to be developed into antistaphylococcal agents.

## 6. Recommendation

Further investigation is ongoing to relate these findings with individual constituents in order to determine the chemical identity of the bioactive compound which is responsible to the antimicrobial activity. In addition to this, morphological and ultrastructural analysis using scanning electron microscope and transmission electron microscope is recommended to confirm the exact mechanism of action and effect* C. odontophyllum* leaf extracts in combination with the standard antibiotics.

## Figures and Tables

**Table 1:** Minimum Inhibitory Concentration (MIC) and Minimum Bactericidal Concentration (MBC) values of *C. odontophyllum* Miq. leaf extracts and antimicrobial agents against MRSA ATCC 33591.

Compound	MIC (*μ*g/mL)	MBC (*μ*g/mL)
*C. odontophyllum* leaf extracts		
Methanol	312.5	625.0
Acetone	156.3	156.3

Antibiotics		
Oxacillin	31.25	31.25
Vancomycin	0.98	0.98
Linezolid	1.56	12.5

**Table 2:** FIC Indices for *C. odontophyllum* leaf extracts in combination with selected antimicrobial agents against MRSA ATCC 33591.

MIC values (*μ*g/mL) in combination				Individual FIC values		FIC index
Antibiotic (A)	MIC A	*C. odontophyllum* leaf extract (B)	MIC B	FIC A	FIC B	
Oxacillin	3.91	Methanol	39.06	0.125	0.125	0.25
	3.91	Acetone	19.53	0.125	0.25	0.375

Vancomycin	0.061	Methanol	312.5	0.0625	1.0	1.0625
	0.061	Acetone	156.3	0.0625	1.0	1.0625

Linezolid	0.39	Methanol	78.13	0.25	0.25	0.5
	0.39	Acetone	39.06	0.25	0.25	0.5

FIC index ≤ 0.5 is interpreted as synergism; 0.5 ≤ FIC index ≤ 2.0 is interpreted as additivity.
